# Transient electrocardiographic changes following smoking cannabis

**DOI:** 10.1002/joa3.12271

**Published:** 2019-11-29

**Authors:** Giacomo Mugnai, Cristina Longo, Chiara Zaltron, Francesca Prevedello, Paolo Segalina, Claudio Bilato

**Affiliations:** ^1^ Division of Cardiology West Vicenza General Hospitals Vicenza Italy; ^2^ Emergency Department West Vicenza General Hospitals Vicenza Italy

**Keywords:** cannabis, ECG, repolarization abnormalities, sinus tachycardia, sympathetic hyperactivity

## Abstract

We report a case of transient ECG abnormalities (negative T waves in the inferior leads) associated with presyncope related to acute cannabis consumption; after a few hours the ECG returned normal. Although pathophysiological mechanisms are not clear, it might be hypothesize a mismatch between increased oxygen demand and decreased oxygen supply or a marked hyperactivation of the sympathetic nervous system.

## Graphical abstract

Cannabis‐related ECG modifications

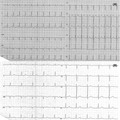

## 1 INTRODUCTION

Cannabis is produced from the *Cannabis sativa*, and includes δ‐9‐tetrahydrocannabinol (THC) and other cannabinoids. It is the most common psychoactive drug used worldwide and it is considered to have low toxicity. However, several cannabis‐related cardiovascular complications have been reported such as tachyarrhythmias, transient ECG abnormalities, acute coronary syndromes including myocardial infarction, cardiac arrest, and asystole^1^, ^2^, ^3^. We report a case of transient ECG abnormalities (negative T waves in the inferior leads) associated with dizziness and presyncope related to acute cannabis consumption.

## 2 CASE REPORT

**Figure 1:**
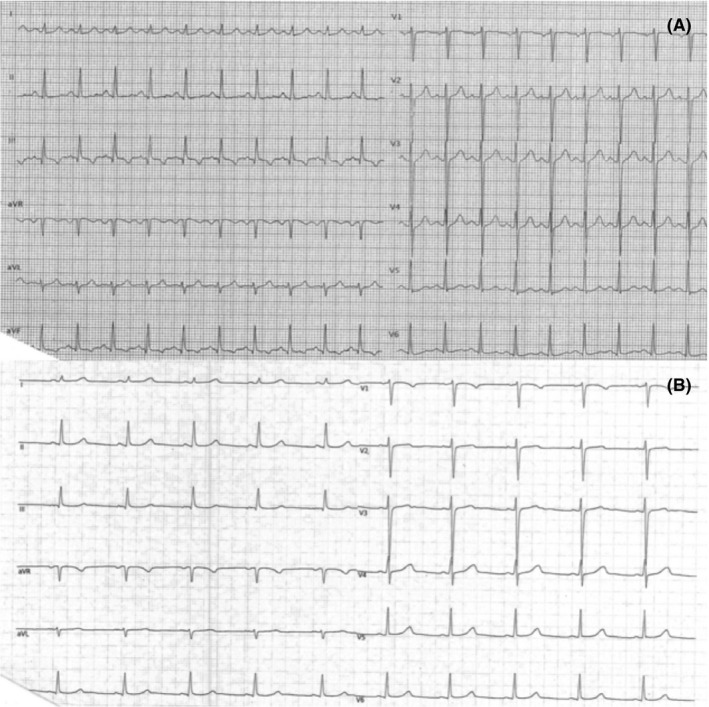
A**,** the ECG shows sinus tachycardia at 126 beats per minute and repolarization abnormalities in the inferior leads. B, the ECG was repeated 12 hours later and presented a normal heart rate and the resumption of a normal repolarization in the inferior leads

A 16‐year‐old female was admitted to the emergency room unit because of marked dizziness and presyncope after smoking cannabis. Particularly, the patient declared she had shared a couple of joints with her friends. She had no history of cardiac diseases and did not show any risk factors for ischemic heart disease. On admission, she was hemodynamically stable and her physical examination was unremarkable; the blood pressure was 125/70 mm Hg and respiratory rate was 18 per minute. The ECG (Figure 1A) showed sinus tachycardia with a heart rate of 126 bpm and repolarization abnormalities in the inferior leads (especially III and aVF). Laboratory investigations showed normal values of hemoglobin, platelet count, electrolytes, blood glucose level, C‐reactive protein, and renal/hepatic function; troponin T was not elevated (4 ng/l; normal values: <17 ng/l)). Echocardiography examination was normal. Parenteral hydration was started and the patient was placed on ECG monitoring. After 12 hours of clinical observation, a prompt relief of symptoms was documented. No atrial tachyarrhythmias, premature ventricular contractions, or atrioventricular blocks were observed on ECG monitoring. A second ECG was obtained 12 hours later documenting a lower heart rate and the complete disappearance of the repolarization abnormalities (Figure 1B).

## 3 DISCUSSION

It is already known that smoking cannabis leads to sinus tachycardia with a dose‐related mechanism. In addition, atrial arrhythmias and other cardiovascular conditions such as acute coronary syndromes, asystole, coronary vasospasm, pseudo‐Wellens’ ECG patterns have been reported after cannabis use^1^, ^2^, ^3^. The cardiovascular effects of cannabis are largely related to its biphasic effect on the autonomic nervous system. At low‐moderate doses, the drug leads to an increase in sympathetic stimulation and a reduction in parasympathetic activity, producing tachycardia and increased cardiac output and blood pressure^1^. On the other hand at higher doses, sympathetic activity is inhibited and bradycardia with hypotension can be observed. Cannabis use has also been associated with premature ventricular contractions, and other reversible ECG changes affecting the P and T waves as well. However, it is controversial if these changes are related to drug ingestion per se or secondary to the increased sympathetic activity and consequent hemodynamic effects^2^.

The present case shows marked sinus tachycardia associated with negative T waves only in the inferior leads; after a few hours the heart rate slowed down and the repolarization abnormalities disappeared. The pathophysiological mechanism is not clear. We might hypothesize a mismatch between increased oxygen demand (because of the increased heart rate and blood pressure) and decreased oxygen supply (because of the increase in carboxyhemoglobin). This mechanism, however, seems less likely considering the age of the patient, the unremarkable clinical history and the absence of cardiovascular risk factors. To our opinion, it appears more suggestive the marked hyperactivation of the sympathetic nervous system with secondary T wave alterations. A paroxysmal sympathetic hyperactivity is known to be associated with sinus tachycardia, T wave inversion, and even ventricular arrhythmias^4^.

In the present case, a coronary artery spasm was excluded because of the absence of ST elevation, normal cardiac enzymes, and lack of typical chest pain. No signs suggesting transmural myocardial ischemia could be observed.

A careful recreational drug history should therefore always be investigated, especially in the presence of unusual cardiovascular presentations and altered mental states.

## CONFLICTS OF INTEREST

None declared.
